# Education: Standards Vary for EH Curricula

**Published:** 2006-10

**Authors:** Kris Freeman

Environmental health science is getting short shrift in some K–12 schools, according to an analysis in the May 2006 *Journal of Geoscience Education*. Students in some states “study the air, water, rocks, plants, and animals, but don’t study any object or process caused by humans. In other states, human–environment (H-E) interactions are shoved into all sorts of nooks and crannies in the science standards,” says lead author Kim Kastens, a senior research scientist at Lamont-Doherty Earth Observatory of Columbia University.

Kastens and Margaret Turrin, education coordinator at Lamont-Doherty, examined science education standards in 49 states (Iowa has no statewide standards), focusing on courses required for graduation. All state standards included at least minimal discussion of H-E interactions, but 15 state standards included less than 1 discussion of H-E topics a year, on average. Only 2 states averaged more than 5 discussions a year.

State curricula more often included information on how humans affect the environment than on how the environment affects humans and human society. Curricula were least likely to include information on ways the actions and decisions of individuals in their daily lives impact the environment; only 57% of state standards included such information.

The paucity of H-E information is at odds with the National Science Education Standards developed by the NAS, says Bora Simmons, director of the National Project for Excellence in Environmental Education of the North American Association of Environmental Educators. Indeed, it counters surveys conducted by Roper Reports/NOP World for the National Environmental Education & Training Foundation showing that 95% of adults and 96% of parents support teaching children about the environment. This general support for H-E education may not be reflected in some standards because of concerns that such topics could generate controversy, says Simmons. “Many standards have not one word on global warming,” adds Kastens.

Kastens and Turrin did find H-E information in standards for other disciplines including health, geography, and consumer studies. For example, in some states children are taught that recycling is healthy, says Kastens, but they don’t necessarily learn the scientific reasoning behind such assertions—information that could well be taught in science class.

The analysis calls for more and better-integrated H-E education so students can build their understanding of environmentally sustainable choices and actions. Kastens says, “An education system that never asks students to think about the impact of their actions on the environment now and in the future is a flawed system.”

## Figures and Tables

**Figure:**
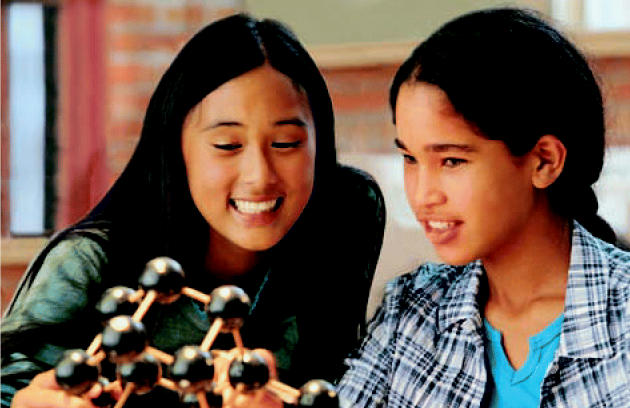
Not making the grade A study of state science education standards shows that students often are not being taught the connection between environment and health.

